# Narcotics information management system in South Korea: system development and innovation

**DOI:** 10.1186/s12913-023-09060-z

**Published:** 2023-01-24

**Authors:** Sang-Yoon Kim, Nam-Wook Cho, Myung-Sik Yoo, Soon-Young Han, Jeong-Wyan Oh

**Affiliations:** 1 Korea Institute of Drug Safety & Risk Management, 5Th Fl., 30, Burim-Ro 169 Beon-Gil, Dongan-Gu, Anyang-Si, Gyeonggi-Do Republic of Korea; 2 Department of Industrial Information Systems, Seoul National University of Science and Technology Graduate School of Public Policy and Information Technology, 232 Gongneung-Ro, Nowon-Gu, Seoul, 139-743 Republic of Korea

**Keywords:** Drug safety, Medical narcotics monitoring, Narcotics information management system, Controlled substance, Medical narcotic drug

## Abstract

**Background:**

As the misuse and abuse of medical narcotics are increasing in South Korea, an information system for the integrated information management of medical narcotic drugs across the nation is needed. This paper presents the development process of the Narcotics Information Management System (NIMS) for the monitoring of medical narcotics usage and the results of its implementation.

**Methods:**

As the NIMS enforces that all narcotics handlers digitally report all information on handling medical narcotic drugs, the functional requirements of the NIMS have been identified in accordance with the Narcotics Control Act. In addition to the functional requirements, the non-functional requirements of the NIMS have been elicited by major narcotics handlers and their associations. The non-functional requirements include privacy, availability, connectivity, interoperability, and data integrity. The system design with entity-relationship diagrams and its implementation processes have been presented.

**Results:**

The NIMS encompasses all narcotic handlers, which comprise exporting, importing, and pharmaceutical companies; wholesalers; hospitals and clinics; and pharmacies, collecting over 120 million cases annually. It enables transparent monitoring throughout the life cycle, from manufacturing, sales, purchase, and disposal of narcotics. As a result, the number of prescriptions for medical narcotics has been reduced by 9.2%.

**Conclusions:**

To the best of our knowledge, the NIMS is the world's first system to manage all information on the total life cycle of medical narcotics, including imports, production, distribution, use, and disposal of drugs. This system has enabled the safety management and monitoring of medical narcotic drugs. Additionally, it provides consistent and transparent information to physicians and patients, leading to the autonomous safety management of narcotics. The successful development of the NIMS can provide guidelines for implementing a narcotics management system in other countries.

## Background

### Rationale

Medical narcotics are essential for treating diseases and alleviating pain, but they are also associated with the risk of misuse/abuse and addiction. Specifically, in the case of synthetic opioid analgesics used for medical purposes, the rates of abuse and addiction are increasing [1]. In the U.S., the number of fatalities due to the overuse of synthetic opioid analgesics, such as fentanyl, increased by 10% in 2018 [2]. Especially during the COVID-19 pandemic, drug abuse in entertainment facilities has rapidly decreased, and the supply of heroin has reduced, whereas abuse of fentanyl and similar substances that can be obtained relatively easily is increasing. Moreover, with the development of the Internet, the illegal or excessive use of medical narcotics(including psychotropic substances and cannabis), such as propofol,^1^ zolpidem, and appetite suppressants, has become more prevalent [3, 4]. Healthcare Information Technology (HIT) is implemented by several countries to facilitate the safe use of medical narcotic drugs and monitor cases of illegal use and misuse/abuse. A number of studies on HIT show that it is an effective tool to control doctor shopping, resulting in a 7% reduction in doctor shopping and a 5% decrease in the fatality rate [5].

### System trends by country

In the U.S., the number of deaths caused by the overdose of narcotic analgesics has been rapidly increasing [6]. Accordingly, to regulate the use of narcotic drugs, controlled substances are designated to Schedule I–V under the Controlled Substance Act, which specifies the registration of handlers and the obligation for the retention of drug handling records. In addition, the Prescription Drug Monitoring Program (PDMP) was established to prevent drug abuse and addiction under the Model Prescription Monitoring Program Act [7]. Fifty states in the U.S. participate in the program, which is operated by each state depending on the local context. The drugs in focus vary by state, but they mainly correspond to controlled substances from schedules II to IV. The prescribers must report the prescription and dispensing information of the drug as well as patient information to the PDMP within 24 h. In the U.S., it has been reported that the implementation of PDMP reduced the prescription of opioids and narcotic analgesics [8, 9]. Moreover, the PDMP report indicated that the number of cases suspected of doctor shopping decreased by 44% [10]. Freeman et al*.* [11] conducted investigative interviews to determine the status of the use of PDMP and pharmacists' awareness. Pharmacists responded that PDMP was very useful in identifying patients who had prescriptions for other narcotics and making dispensing decisions.

In Canada, controlled substances are classified into Schedules I–V under the Controlled Drugs and Substances Act [12]. Since the Narcotics Safety and Awareness Act was implemented in 2010, seven provinces introduced a computing system for monitoring the prescriptions for narcotics [13], and New Brunswick and Prince Edward Island are developing an information system. In Ontario, misuse of synthetic opioid analgesics, benzodiazepines, and stimulants decreased, owing to legislations related to narcotics and the Narcotics Monitoring System (NMS) [14].

Australia has classified medical narcotics into schedule 8 according to the Standard for the Uniform Scheduling of Medicines and Poisons, also known as the Poisons Standard [15], and has introduced the Electronic Recording and Reporting of Controlled Drugs initiative to prevent the abuse of controlled substances. Accordingly, the state of Tasmania operates the Drug and Poison Information System Online Remote Access (DORA) to manage the prescriptions for narcotics and poisonous substances [16]. Furthermore, data on the use of controlled substances are maintained on My Health Record, which is self-managed by the patients as per the My Health Records Act [17, 18]. According to the 2018 Australian Annual Drug Overdose Report, the fatality rate from addiction to prescription synthetic opioid analgesics in Tasmania decreased by approximately 27% compared with the national average between 2012 and 2016 since DORA was introduced in 2009 [19]. Boyles [16] analyzed the fatality rate due to overdose of synthetic opioid analgesics after the introduction of DORA, which showed a significant reduction in the states. This finding demonstrates that DORA is a useful tool for reducing the fatality rates related to the use of narcotic drugs.

In Korea, public insurance is provided to the entire population to receive care in a hospital. However, if they did not use public insurance, regulatory authorities would be unable to track their use of medical narcotics. In addition, forged and illegal prescriptions or excessive prescriptions for narcotic drugs are some of the causes of social problems. The number of narcotics users increased by 12.5% in 2020 compared with 2019, and in particular, medical narcotic drugs, including "fatigue recovery injections (propofol)," "weight-loss drugs (anorectic agents)," "date rape drugs (sleep aids, muscle relaxers)," and "study drugs (stimulants)," are being overused or misused; however, an integrated information system for medical narcotics is still lacking.

Following the development of the Narcotics Information Management System (NIMS)^2^ by the Korean government in 2014 for the systematic management and monitoring of medical narcotics, a 2-year pilot project was launched. Since then, for medical practitioners handling medical narcotics, the medical narcotics handling reporting system has changed from a written recording system on the management register to an electronic reporting system via NIMS, with the new system taking effect in May 2018.

## Methods

### Institutional information

The objective of the NIMS is to ensure that all narcotics handlers digitally report all information on handling medical narcotic drugs. However, in Korea, the narcotic usage per prescription for situations not covered by public health insurance, such as treatments for beauty, plastic surgery, and health promotion, and cases of psychiatric illnesses where patients do not wish to disclose details, has not been properly controlled. Therefore, it is necessary that the NIMS monitor all the cases of medical narcotics handling information regardless of whether or not they claim benefits from the national medical insurance system. Medical narcotic handlers should be obliged to report via the NIMS pursuant to the Narcotics Control Act. To this end, NIMS supports pharmaceutical companies, wholesalers, hospitals, clinics, and pharmacies, which are included in automatic reporting and interactions via software and computing systems, such as medical record (EMR), enterprise resource planning (ERP), and claims/prescriptions software. Kruse et al*.* (2020) reported that increased interaction, whether voluntary or mandatory, between systems leads to more successful use of HIT [5]

The report on narcotic usage includes the date, product name, quantity, etc., and while a serial number should be reported for drugs, a manufacturing number should also be reported for psychotropic drugs. Drug handling medical business operators and drug handling retailers shall report the patient's resident registration number, disease classification symbol, and doctor's license number. However, since the drug handling reporting system is for managing actual medical drugs, it does not report a narcotics handling case if only a prescription has been issued without the direct preparation and administration of the narcotics. Additionally, exceptions to correct serial or manufacturing numbers are allowed for treatments in emergency rooms.

To the best of our knowledge, the NIMS is the first system in the world to manage all information on the total life cycle of medical narcotics from import, production, distribution, use, and disposal of those drugs, as well as a unique product identification code, doctor's unique identification number (license number), and patient's unique identification code (resident registration number).

The Korea Institute of Drug Safety & Risk Management has been designated as the Center for Narcotics Information Management to operate the NIMS pursuant to Article 11 (2)^3^ of the Narcotics Control Act and Article 8 of the Enforcement Decree of the Act. The Center for Narcotics Information Management offers services related to the establishment and implementation of plans for collecting, investigating, using, and providing information on narcotics in addition to training and promotion, research and investigation, establishment and operation of the system, and provision and utilization of information.

### System requirements

#### Functional requirements

The functional requirements of the NIMS have been identified in accordance with the Narcotics Control Act. A narcotics handler or a person approved to handle narcotics shall report to the Minister of Food and Drug Safety on matters regarding the name, quantity, date of use, place of purchase, inventory, and serial numbers of narcotic drugs that are exported, imported, manufactured, sold, received by transfer, transferred, purchased, used, discarded, dispensed, administered, provided for administration, or used for academic research, and the name of each party that was included in all such transactions. In such cases, if a narcotics handler or a person approved to handle narcotics is the other party involved in the handling of narcotics, the extent of handling and permission or approval number or date should be reported together with the aforementioned report. A medical trader or retailer must enter the patient registration number, disease classification code, name of the business, and name and license number of the doctor issuing prescription. If a veterinarian uses narcotics to treat animals, the resident registration number of the owner or manager of the animals must be recorded.

**Fig. 1:**
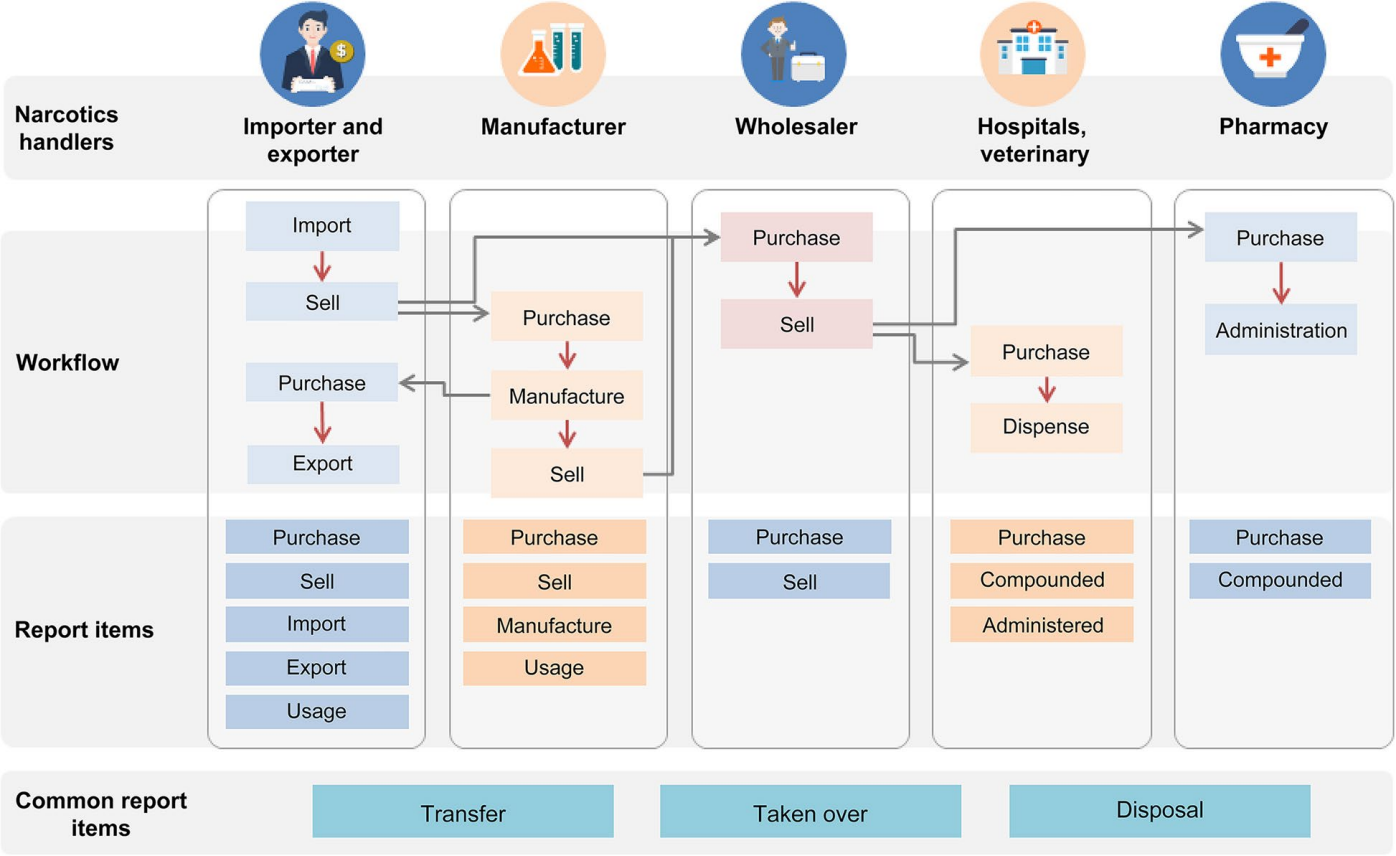
Workflow of the narcotics information management system. From the production to disposal of the medical narcotics reporting process

The management process of medical narcotics based on the information above is shown in Fig. 1. Narcotics handlers should report information corresponding to the Report Items or Common Report Items given in each workflow step using the NIMS.

#### Non-functional requirements

In addition to the functional requirements, the non-functional requirements of the NIMS have been elicited by major narcotics handlers and their associations, including the Korean Medical Association, Korean Pharmaceutical Association, Korean Society of Health-system Pharmacists, Korean Veterinary Medical Association, Korea Pharmaceutical Distribution Association, Korea Pharmaceutical and Bio-Pharma Manufacturers Association, and Korean Research-based Pharmaceutical Industry Association. Their needs were identified via a series of meetings and interviews, and potential conflicts were coordinated, which minimized confusion at an early stage of the implementation of the system by specifying the requirements. For example, special attention has been paid to the different characteristics of medical specialists.

#### Privacy

The first non-functional requirement identified during the process was privacy. For example, the association of psychiatric practitioners had concerns that if the prescription history is reported, patients might evade medical consultations. To resolve the issue, we used the asymmetric encoding algorithm of the Rivest–Shamir–Adleman method to protect personal information and security measures such, as Secure Socket Layer, in the communication pathway.

#### Availability

The second non-functional requirement was availability. Industry representatives wanted some information to be entered into the system automatically, such as the basic information of patients. The autocomplete function fulfilled this request. Moreover, there was another request for a function that would allow checking stocks by items, transfer of the stock, or compensation of the stock. The function of reporting and stock transfer, storing, and releasing has been implemented as a response to the request.

#### Connectivity

As the NIMS is computationally connected to the software used by the narcotics handlers (EMR, ERP, claims, and prescription software), connectivity was identified as another non-functional requirement. The connecting system was designed for users who are not familiar with computer systems or elderly people to generate a report automatically via the software so as to increase the capacity for computational reporting.

During connected reporting, narcotics handlers can provide information via the software used by them without entering the NIMS web system. At an early stage of designing the connecting system, a difficulty arose with regard to the exact status of the software companies used by the narcotics handlers. Therefore, efforts have been made to identify the status via cooperation with relevant associations, such as physician associations, official documents, meetings, and information sessions.

#### Interoperability

Interoperability for connectivity was also considered. There was difficulty in persuading the companies because the languages and structures developed by each software varied, and a design for standardization was important. To that end, a consultative group was formed including general hospitals, clinics, and software companies. Based on their requirements, the layout of the connecting system was designed, and a connection development guide and sample source codes for connection in five major languages (Java, C#, Visual Basic, Delphi, and PowerBuilder) were provided. Then, the software companies were invited to information sessions and meetings, and they were divided into groups of pharmaceutical companies, wholesalers, general hospitals, hospitals or clinics, and pharmacies.

#### Data integrity

As the NIMS deals with narcotics handling information from more than 0.5 million cases a day from various narcotics handlers, data integrity and data quality management are of critical importance. If data integrity is not secured, there is a risk of data forgery and falsification, statistical errors, and the safety management of narcotics being jeopardized. The NIMS was not designed to be used to arbitrarily modify or delete all narcotics handling records reported on the NIMS. It was designed to trace the history, which is connected to the history of existing records in the form of chains, in the event that the reported information was modified. In accordance with the policy, all data is backed up daily or weekly. In addition, the database is only accessible through the access control system, and the entire history of instruction execution is recorded and backed up in a separate log. The process for data quality management is described in the System Verification section.

Other requirements include system improvement, such as facilitating administrative disposition, and non-functional requirements, such as training support.

**Fig. 2:**
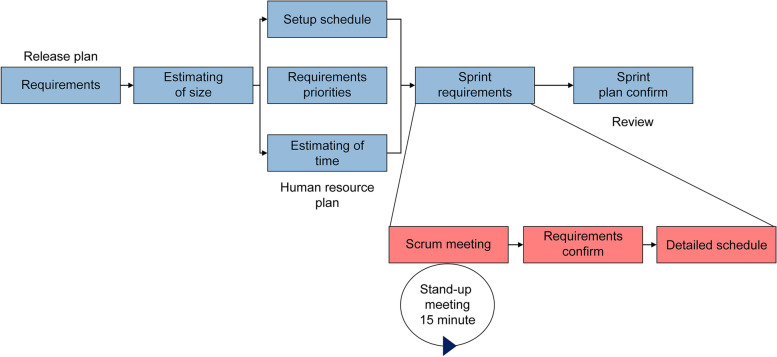
Development process: The development process was tailored using the Scrum development methodology of agile

As shown in Fig. 2, each step of the development process was tailored using the Agile Scrum methodology. Project leaders, such as project managers, developers, and database administrators who participated in the NIMS development project, attended and shared the review of the previous day's work as well as the work plan for that day. After the entire project schedule was established, it was segmented into sprints, and Scrum meetings took place every morning for 15 min. Shared information was visualized via Post-it and Redmine. This process was helpful in identifying issues among project participants early on and sharing the progress.

The development outputs were tailored based on the information engineering methodology, and during the analysis of the requirements, the requirement specifications and traceability matrices were prepared. This process facilitated securing traceability for the final functions.

### System design

**Fig. 3:**
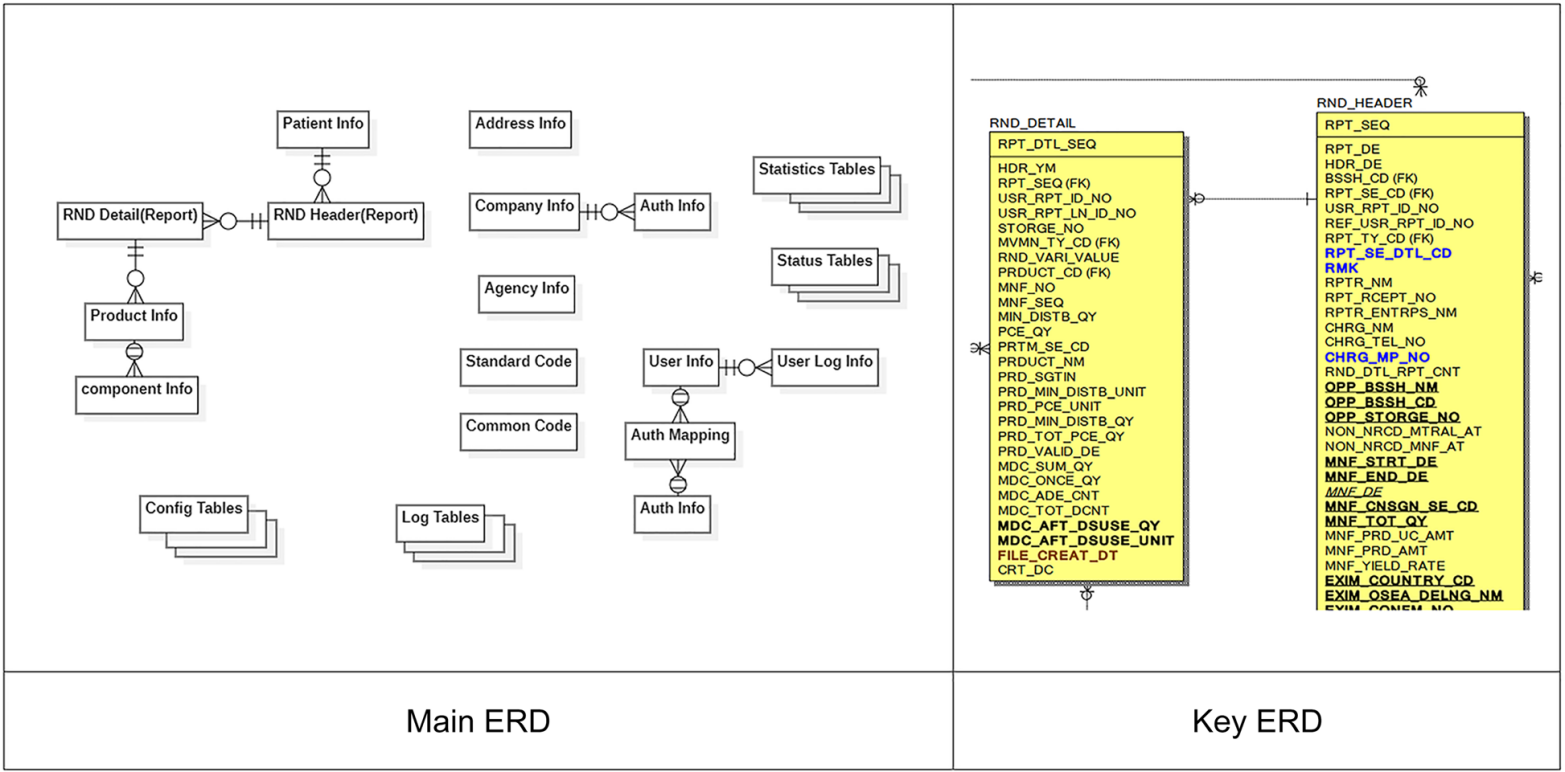
Entity-relationship diagram of the narcotics information management system. The database schema was designed with master-detail structure

The functional specifications were prepared based on the requirements to design a layout and create a database entity-relationship diagram for each handler. Particularly, the database was normalized by each of the first services for handling narcotics and considering the various workflows of the narcotics handlers; the master-detail structure was designed to meet the performance requirements via denormalization. For example, prescriptions are saved in RND_Header, and a list of narcotic medications according to the prescriptions is saved in RND_Detail, as shown in Fig. 3. This structure was designed to be applied to all narcotics handling procedures in common, from production to disposal, regardless of the stages of prescription and preparation for drugs. It was designed so that the primary key is generated by the users in the user field and not by the system. This was intended to speed up the "create, read, update, and delete" operations in the database while connecting to various systems and allowing the users to modify and delete information on medical narcotics handling with their primary key. If users modify or delete data, the history is not deleted in the database, and the modification history can be traced by connecting the original record to the modified record in the form of a chain.

**Fig. 4:**
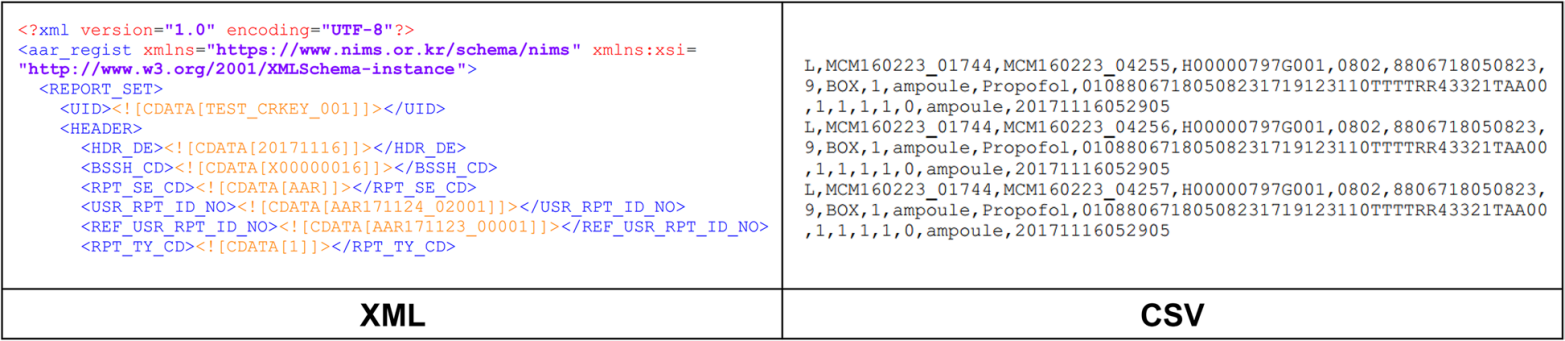
Report file format. The reporting file formats were designed in.xml and.csv

The NIMS provides connections via an HTTP-based representational state transfer application programming interface (RESTful API) and service-oriented architecture-based enterprise service bus (ESB). Moreover, a reporting file format can be chosen between extensible markup language and comma-separated values, as shown in Fig. 4, which is a data format required by global pharmaceutical companies. It was designed in such a way that if it fails to report owing to problems with the network or connecting software, it generates a report by uploading the reporting files.

### System development

The electronic government framework was formed based on Java's Spring Framework, which should be prioritized when establishing websites for the government, public institutions, and public enterprises according to the guidance of the Ministry of the Interior and Safety in Korea. The NIMS was developed based on the Korean electronic government standard framework and activated in Java Development Kit 1.7. The NIMS website and its connecting system were designed as a three-tier structure where the Web Server uses Apache 2.2.15, the WebSphere Application Server uses Weblogic 12c. Oracle Exadata, an appliance device, and the database uses Oracle 12c.

The connecting system was developed in the RESTful API method and ESB. To deal with the initial simultaneous transactions, it was implemented in an asynchronous pattern, followed by a two-step method to confirm the completion after sending the reporting information. However, a synchronous model was provided after the system was stabilized. Currently, the synchronous method processes 80–120 transactions per second for which resources are used on average within 10–20%.

Infrastructure flexibility was secured via a virtualized server, and the network interval within the system was formed to be 1G, while the interval between the web application server and the database was formed to be 10G, where a large number of arithmetic operations are performed.

### System verification

Upon the completion of the system development, a pilot test was performed from March to May 2018. During the test, a meeting was organized for the major users that led to improvements in user inconvenience, e.g., user interface and user experience.

Since the NIMS deals with narcotic reports of more than 0.5 million cases a day, data quality management is a critical issue. To that end, a database quality management (DQM) process has been formed to verify user input data during the test. As a result, data integrity issues are classified into the following categories: duplicate entry, formation error, logic error, and the subject of surveillance. For any system errors, the corresponding function was modified. Users are notified about simple user errors via email or mail. If the errors are assumed to be intentional or repetitive, they are classified as the subject of surveillance to be monitored, and appropriate information is sent the Ministry of Food and Drug Safety so that they can provide guidance and conduct supervision.

In a study by Jung Seung-Ho et al*.* [20] (recited from National Information Society Agency Report, Korea 2011), the average error rate of data that belonged to administrative and public institutions was 5.19%, which was found to be higher than that of private sectors. However, the DQM system managed to reduce the error rate from 5% at an early stage to 0.05% via the DQM system.

### Data verification and Utilization of the system

**Fig. 5:**
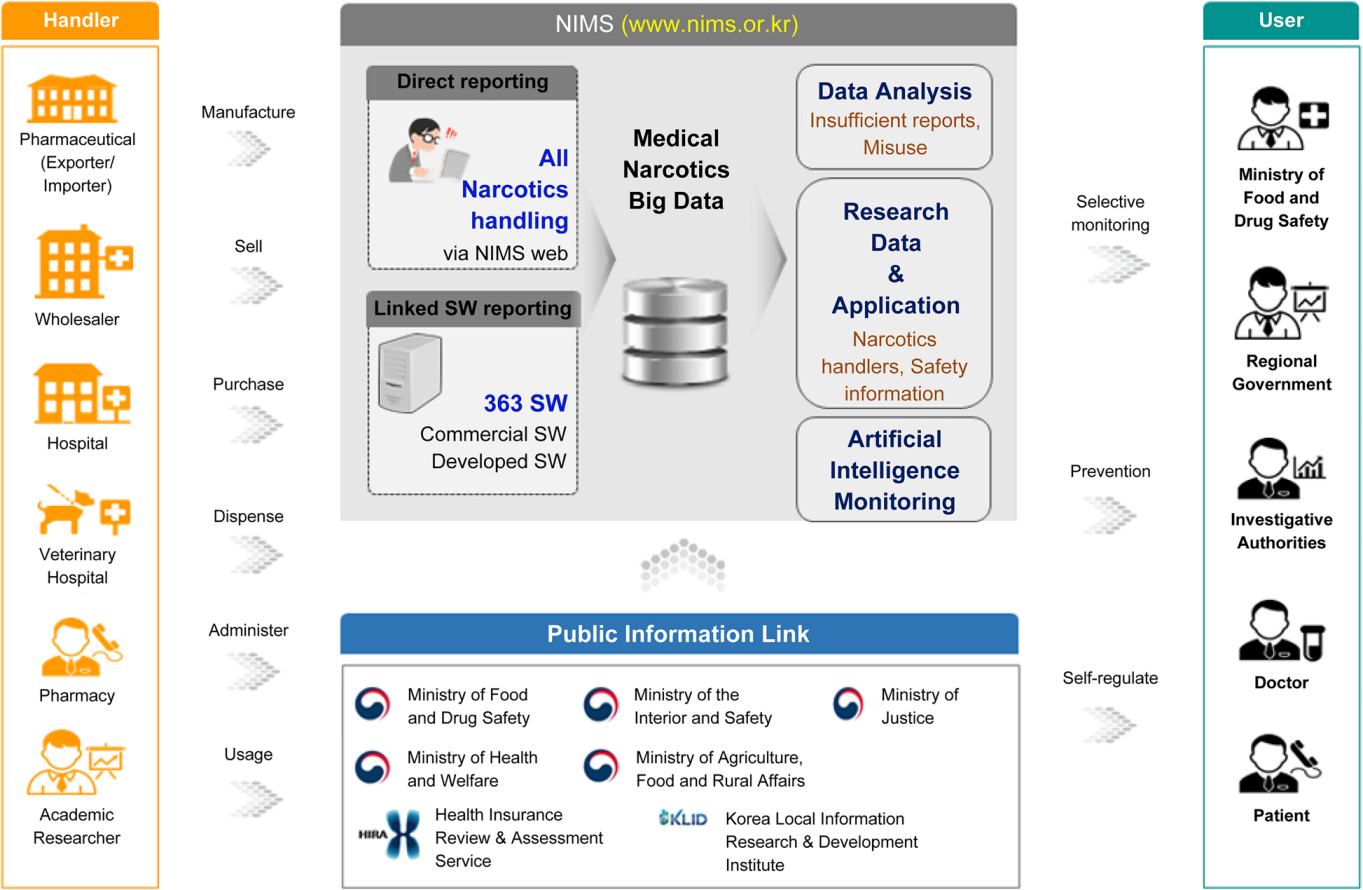
Structure of the narcotics information management system. The enterprise architecture for reporting systems

Since the implementation of the NIMS in May 2018, narcotics handlers can check their inventory and details of use via the NIMS, therefore, replacing the narcotics handling management register. Additionally, abnormal narcotic usage is assessed via cross-analysis using the database itself and connection with the public information in seven government institutions. The analyzed information is provided to the Ministry of Food and Drug Safety and relevant administrative institutions so that they can conduct intensive screening inspections and monitoring. The enterprise architecture is shown in Fig. 5.

## Results

### Registration status

Since the introduction of NIMS in May 2018, a total of 63,311 narcotics handlers have been registered as members of NIMS, with a total of 115,889 approved cases in South Korea from May 2018 to November 2021. In Korea, when dealing with medical narcotics, narcotics handlers must register and report to NIMS, except for those who do not handle medical narcotics or just issue a prescription to a patient without handling narcotic substances. The number of registered members compared with approved cases for each type of narcotics handler is shown in Table 1.

**Table 1:** Approved cases of narcotic handling vs Number of NIMS members

Classification	Approved as narcotic handler cases, n	NIMS members, n	NIMS membership rates, %
Sum	115889	63311	55%
Manufacturer	74	94	127%
Importer and exporter	70	95	136%
Raw material user	94	105	112%
Wholesaler	1985	2703	136%
Pharmacy	24151	26862	111%
Hospital	80372	27250	34%
Academic researcher	1569	2077	132%
Other handling approvers	7574	4125	54%

Note that a membership rate of more than 100% reflects the institutions whose license/approval was canceled or which do not handle medical narcotics anymore but are still maintaining their membership in the NIMS for the purpose of individual narcotics handling records. Additionally, hospitals have lower membership rates because medical practitioners are not supposed to report to NIMS if they do not handle actual substances but issue prescriptions only. During this period, data pertaining to a total of 405 million cases of narcotic drug handling were collected, and on average, data pertaining to approximately 0.5 million cases of narcotic drug handling are being collected every day.

### Operational results

The operational status of the NIMS in 2020 is shown in Table 2. The most frequent type of narcotics handling information was drug disposal in hospitals, clinics, and pharmacies at 62.5%, followed by reporting of administration in hospitals at 26.5%.

**Table 2:** Number of cases by report type of narcotics handling information (number of reported cases in 2020)

Type of report	Cases	Rates(%)
Dispensing	77534810	62.54%
Administration	32916453	26.55%
Purchase	5790677	4.67%
Sale	5053226	4.08%
Storage transfer	2351957	1.90%
Other released products	80523	0.06%
Other stored products	79831	0.06%
Takeover	51082	0.04%
Handover	43398	0.04%
Disposal	36437	0.03%
Use	27880	0.02%
Manufacturing	3760	0.00%
Import	1308	0.00%
Use of raw materials	1267	0.00%
Export	180	0.00%
Consigned products stored	79	0.00%
Consigned products released	52	0.00%
Sum	**123972920**	**100.00%**

The reports of the NIMS from January to December 2020 were analyzed, and it was found that 17.47 million people out of the entire population of 51.83 million inhabitants of South Korea used medical narcotics, which is 33.7% of its population. It should be noted that patients used for medical examination purposes are included. A total of 9.94 million women (57.2%) and 7.44 million men (42.8%) used medical narcotics. In other words, 14.4% more women used this type of drug.

People aged between 40 and 60 years were predominant, out of which those in their 50 s used medical narcotics most commonly at 21.2%. Other percentages with regard to age categories were as follows: 40 s, 19.3%; 60 s, 18.3%; 30 s, 13.1%; 70 s, 10.8%; 20 s, 8.3%; 80 s, 5.1%; ≤ 10 years, 3.2%; and ≥ 90 years, 0.7%.

It was shown that anesthetics/analgesics (used by 9.08 million people) and sedative-hypnotics (8.08 million) were the most commonly used drugs, followed by antianxiety medications (used by 6.03 million people), analgesics (2.96 million), anorectic agents (1.31 million), antiepileptic medications (1.02 million), antitussives (0.6 million), and medications for attention deficit hyperactivity disorder (0.14 million).

Table 3 summarizes the usage of narcotics in Korea. It was reported that 102,662 physicians from 39,629 medical institutions used 1,751,865,742 medical narcotics, which were administered to 17,474,955 patients in 2020 in South Korea. Out of all types of prescribing institutions, clinics used medical narcotics most often.

**Table 3:** Usage of narcotics in 2020 in South Korea

Prescribing institutions (types)	Number of prescribing institutions	Number of prescribing doctors	Number of patients	Number of prescription cases	Used dosage (number of tablets/units)
General hospitals	402	41402	5835456	33175099	454367714
Hospitals	2163	12703	2838206	13916539	146226008
Nursing hospitals	1656	7519	328340	9135045	81207298
Clinics	34748	46713	11288899	43833941	1068183038
Public healthcare centers, etc	660	1468	19160	60179	1881683
Sum	39629	102662	17474955	100120803	1751865742

Seo Young-Seok, an assembly member of the Health and Welfare Committee of the National Assembly of the Republic of Korea, reported that in the case of fentanyl patches—which teenagers get prescriptions for in their name or other person's name for the purpose of inhalation—a total of 0.32 million patients got a prescription and there were 0.97 million prescriptions in the NIMS from July 2020 to April 2021. The number of patients who got prescriptions for the patches in the Drug Utilization Review(DUR) submitted by the Health Insurance Review and Assessment Service was 0.23 million patients, and the number of prescriptions was 0.29 million cases, showing 76% difference [21]. This has been possible because all prescriptions, regardless of insurance coverage, should be reported mandatorily in the NIMS. Therefore, it is important to manage medical narcotics along with prescriptions without insurance coverage.

### Utilization results

Since the implementation the NIMS in 2018, the reported information has been analyzed and utilized in various ways.

The "My Medication History Inquiry" service, which has been in operation since February 2020, allows patients to search their medication records pertaining to administering medical narcotics. According to the "Status of Disciplinary Action Against Teachers Involved in Drug Crimes in the Last 5 Years" by the Ministry of Education in 2020, it was found that more than half of the teachers did not realize that their diet pills were psychotropic drugs [22]. Patients can check their narcotics medication history via "My Medication History Inquiry Service," which can prevent them from the misuse of medical narcotics.

The "Information Network to Prevent Doctor Shopping for Narcotics" function for doctors, which has been in operation since June 2020, checks and prevents the overuse of medical narcotics by providing a patient's history of using medical narcotics in cases of patients visiting the hospital who are suspected of being a drug addict. Disputes could have occurred in the past owing to a doctor's refusal to treat patients, etc., but the Ministry of Food and Drug Safety has recently inserted Article 30 (2) of the Narcotics Control Act for doctors to avoid prescribing medical narcotics to patients when doctor shopping for narcotics is suspected. In addition, via big data analysis of the NIMS, "Guidance for the Safe Use of Medical Narcotics" reports are being sent to doctors who prescribe large amounts of medical narcotics, such as zolpidem, propofol, and anorectics. The "prenotification" service is currently being developed, including a written warning and on-site inspection if safety standards are not met.

**Fig. 6:**
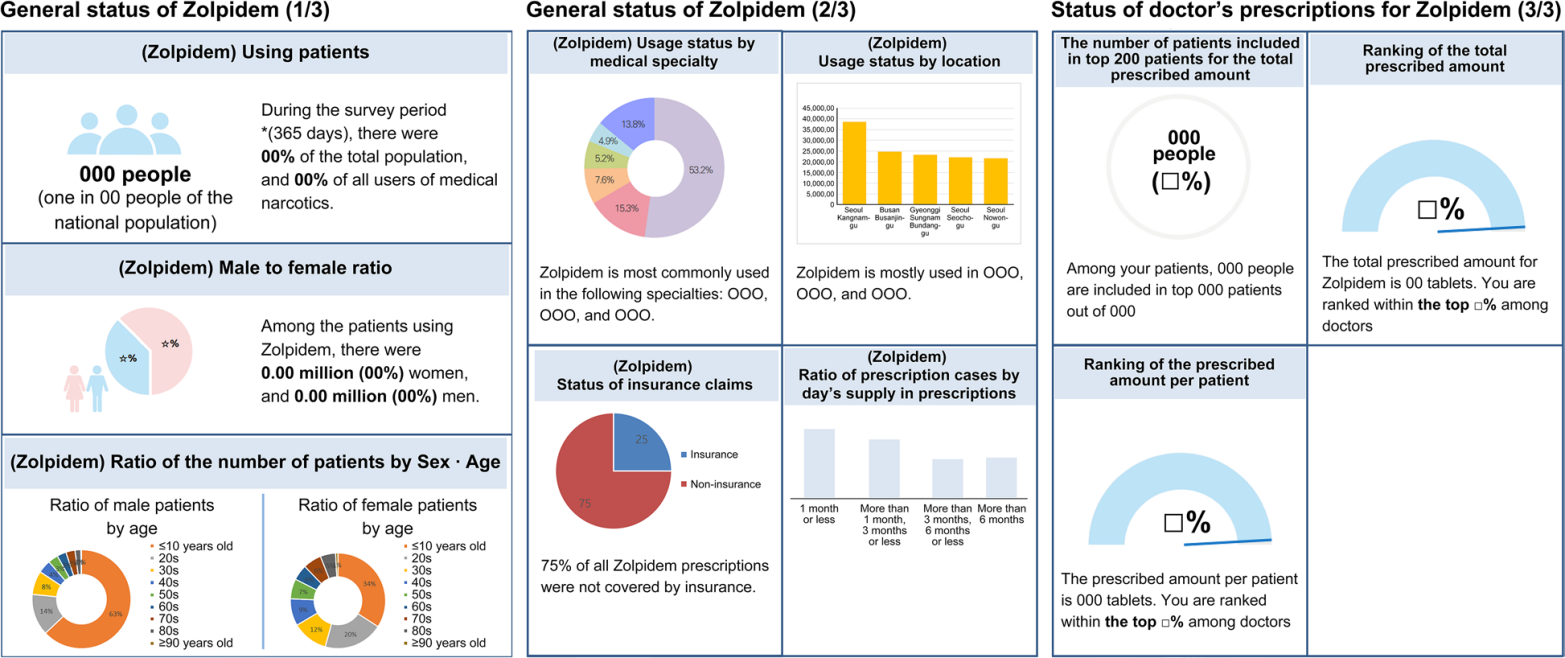
Guidance for the safe use of medical narcotics. The "Guidance for the Safe Use of Medical Narcotics" report sample

In 2019, prescription details were analyzed for three months before and after the receipt of the official letter entitled "Guidance for the Safe Use of Medical Narcotics." Based on data reported through NIMS and analysis of statistical information, official letters were automatically generated on NIMS and sent specifically to each hospital. Figure 6 is a template for an official letter that provides national statistics such as the number of patients who received prescriptions for zolpidem and their gender (as reported on NIMS), regional statistics of the locations of the relevant hospitals, and statistics on prescriptions issued by each hospital.

The results showed that the average amount of propofol, zolpidem, and anorectic prescriptions per patient was reduced by 9.2%. In particular, prescriptions for zolpidem were reduced by 6.8%, propofol by 5.9%, and anorectics by 11%, indicating that it positively affected the appropriate use of narcotics in clinical settings [23]. In addition, a decrease in the average prescribed dosage indicates that practitioners issuing prescriptions are aware of their prescription dosage and are focused on prescribing drugs to patients who require them. This means that the safety of narcotics is being considered at the prescription stage. Moreover, other effective results include investigations requested for suspected cases of over-administration and illegal transfer of medications in hospitals and clinics, forged prescriptions by patients, and patients suspected of using a dead person's name illegally [24, 25]. The "Guidance for the Safe Use of Medical Narcotics," report sample is shown in Fig. 6.

Since July 2020, research data have been open to the public so that data on medical narcotics can be safely used via the "Narcotics usage data open service".

## Discussion

The rates of misuse and abuse of medical narcotic drugs are increasing worldwide. Similarly, in Korea, an increase was observed in the number of cases of forgery and falsification of prescriptions for medical narcotic drugs. To also enhance the safety of the management of medical narcotic drugs, Korea implemented a medical narcotic drug handling reporting system that replaces the manual record management method with a digital system and established an integrated narcotic drug management system for this purpose. To the best of our knowledge, the NIMS is the world's first system that can manage the entire life cycle of a medical narcotic drug, including importing, producing, distributing, using, and disposing of, as well as track the production, doctors who prescribed, and patients who received the medical narcotic drugs across the country.

In this paper, the development process, operation results, and utilization performance for establishing an integrated narcotic drug management system were presented. In the first section, we examined the current status of misuse and abuse of medical narcotic drugs as well as the safety management systems and research results of medical narcotic drugs by country. The existing HITs, such as the PDMP in the U.S., NMS in Canada, and DORA in Australia, were determined to be effective in identifying the prescription status and making orders for dispensing. HIT was also useful in decreasing doctor shopping. In the second section, we assessed the Korean legal system for safety management of narcotic drugs and the process of establishing the integrated narcotic drug management system; requirements analysis, design, development, and test processes were presented. During this process, we collected requirements via user meetings and briefing sessions and established a computer linkage system with ERP, EMR, and billing and prescription software, etc., to adapt to computer reporting. Additionally, a tailored Agile Scrum methodology was utilized for communication between participants and for sharing project progress. In the third section, the data on the use of medical narcotic drugs over one year were analyzed. The results indicated that the use of medical narcotic drugs was relatively greater at the clinic level compared with general hospitals. The number of prescriptions not covered by insurance was > threefold higher than that of prescriptions covered by insurance managed by the government. Utilization services, such as the "Drug Prevention Information Network" and "Medical Drug Safety Helper" for doctors and "My Drug History Inquiry Service" for patients, were introduced to provide data from the integrated narcotic drug management system, and it was confirmed that these utilization services reduce the average prescription per patient. Based on these findings, we conclude that medical safety management via computer systems is useful and efficient.

Despite the overall contributions of the present study, we cannot help admitting the limitations. First, we could not compare the difference before and after the system installation because the narcotic drug handling status was recorded and managed manually by each narcotic drug handler. Second, since the system was in a trial period from 2018 to 2019, the paper only analyzed the data from 2020. Therefore, further analysis of the cumulative data, including age, season, disease, and social issues, would be a suitable future research topic.

## Conclusions

In Korea, the safety management of medical narcotic drugs via the NIMS (a HIT system) resulted in significant results, similar to other countries. We believe that the successful development of the NIMS can provide guidelines for implementing a narcotics management system in other countries. Additionally, it is necessary to efficiently perform narcotic drug safety management via data analysis and seek strategies to establish a social safety net.

## Data Availability

The data used in this paper is not publicly available under Article 11–5 of the Korean Narcotics Control Act. Statistical data can be provided for research, investigation, and education on drug misuse on reasonable request from the corresponding author.

## Abbreviations

DORA: Drug and Poison Information System Online Remote Access
DQM: Database quality management
ERP: Enterprise resource planning
EMR: Electronic medical record
ESB: Enterprise service bus
HIT: Healthcare Information Technology
NIMS: Narcotics Information Management System
NMS: Narcotics Monitoring System
PDMP: Prescription Drug Monitoring Program
RESTful API: Representational state transfer application programming interface

## Footnotes

1 propofol is classified as an antipsychotic drug in Korea.

2 Information system established with functions for narcotics handlers or handling approvers to electronically report all details, including production, distribution, and use of narcotics, and functions of analyzing such information, to stop illegal outward flows and abuse of narcotics, pursuant to Article 11 (Reporting of handling narcotic drugs) of the Narcotics Control Act.

3 Narcotics management system for narcotics handlers including pharmaceutical companies which produce, distribute, and use narcotic drugs, exporting and importing companies, pharmaceutical wholesalers, hospitals and clinics, pharmacies, and academic researchers to report all information including exports and imports, manufacturing, use of raw materials, sale, purchase, preparation, administration, handover, takeover, and disposal in the Narcotics Information Management System (NIMS), pursuant to Article 11 (Reporting of handling narcotic drugs) of the Narcotics Control Act.
